# Heterogeneous polymerization via two-step crosslinking for tunable microribbon hydrogels

**DOI:** 10.1088/1758-5090/ae235a

**Published:** 2025-12-17

**Authors:** Mahsa Karimi, Fereshteh Ahadi, Niloofar Esmati, Mingyue Fan, Lin Han, Christopher Y Li, Li-Hsin Han

**Affiliations:** 1 Department of Mechanical Engineering and Mechanics, Drexel University, 3141 Chestnut St., Philadelphia, PA 19104, United States of America; 2 Department of Material Science and Engineering, Drexel University, 3141 Chestnut St., Philadelphia, PA 19104, United States of America; 3 School of Biomedical Engineering, Science and Health Systems, 3141 Chestnut St., Philadelphia, PA 19104, United States of America

**Keywords:** heterogeneous hydrogel, hydrogel polymerization, multi-step crosslinking, building blocks, porous hydrogel, tissue engineering

## Abstract

Hydrogels are widely used in tissue engineering, but conventional homogeneous polymerization often creates dense matrices that hinder cell migration and restrict extracellular matrix production. The motivation of this project was to overcome these limitations by developing a heterogeneously crosslinkable hydrogel platform that enables both cell migration and matrix deposition. We present a two-step heterogeneous polymerization approach that introduces spatial variations in matrix density, producing tunable, cell-sized pores that promote migration, proliferation, and matrix synthesis. As an implementation, gelatin was pre-assembled into microribbon-like building blocks using a dynamic molding process, methacrylated to introduce crosslinkable groups, chemically modified, washed, and freeze-dried. Upon rehydration, the ribbons formed a moldable paste that could be mixed with cells and photo-crosslinked into scaffolds with *in situ*–formed, cell-sized pores. The main novelty of this method is the introduction of chemical modifications with methacrylic anhydride (MAA), acetic anhydride (AceA), and succinic anhydride (SucA), which enable a controlled two-step heterogeneous polymerization and allow independent tuning of scaffold microstructure, mechanics, and degradation. AceA reduced crosslink density and accelerated degradation, whereas SucA promoted swelling, enhanced mechanical strength, and slowed degradation. Cell studies revealed that SucA-modified scaffolds supported superior adhesion and proliferation compared to AceA-modified and unmodified controls. Such work may significantly impact the design of next-generation scaffolds by providing a versatile platform that integrates structural, mechanical, and biochemical control for regenerative medicine applications.

## 1. Introduction

Hydrogels are widely used in tissue engineering. They are water-rich and can be synthesized from a broad range of synthetic and naturally derived polymers. Hydrogels’ chemical properties and mechanics are tunable by adjusting polymer composition, crosslinking density, and solid content. Encapsulating cells in 3D within a hydrogel that provides appropriate chemical and mechanical properties can promote tissue regeneration, when such components support the cellular activities that drive repair [1, 2]. However, hydrogels crosslinked from standard biopolymers have a major drawback. They are typically formed with sub-micron size meshes that confine cells, limiting spreading, migration, cell–cell contact, and matrix deposition; this confinement often hinder tissue formation and, in some contexts, trigger apoptosis [3]. Degradable or cell-remodelable hydrogels increase the network’s effective mesh size via hydrolysis or cell-secreted enzymes and may improve migration and matrix deposition, but such approach requires finely tuned degradation to maintain hydrogel integrity and depends on cell- and tissue-specific enzymatic activity, which is difficult to control, especially for clinical application [4, 5].

To overcome this limitation, we present a two-step crosslinking strategy. In step one, we shape common biopolymers into cell-scale building blocks. In step two, we crosslink across, and within, these blocks to fuse them into porous hydrogels with controlled, interconnected, cell-scale cavities. These cavities form *in situ* during crosslinking and provide cell mobility without relying on degradation. Cavity geometry is set by the size and shape of the building blocks and can guide cell distribution and organization. We then use post-functionalization to tune the pores’ microstructure, mechanics, swelling, degradation, and interfacial chemistry, and we evaluate early cell attachment and proliferation under these conditions.

Notably, this method supports direct encapsulation of live cells, as in conventional hydrogel systems. In step two, the building blocks, which form an aqueous paste, are mixed with cells, then crosslinked to form a porous hydrogel with interconnected, cell-scale cavities that encapsulate the cells. Compared to traditional hydrogel crosslinking, this two-step method sets a defined internal architecture that permits and organizes cell spreading, proliferation, and matrix deposition [4], while requiring minimal effort in step one.

**Figure 1.**
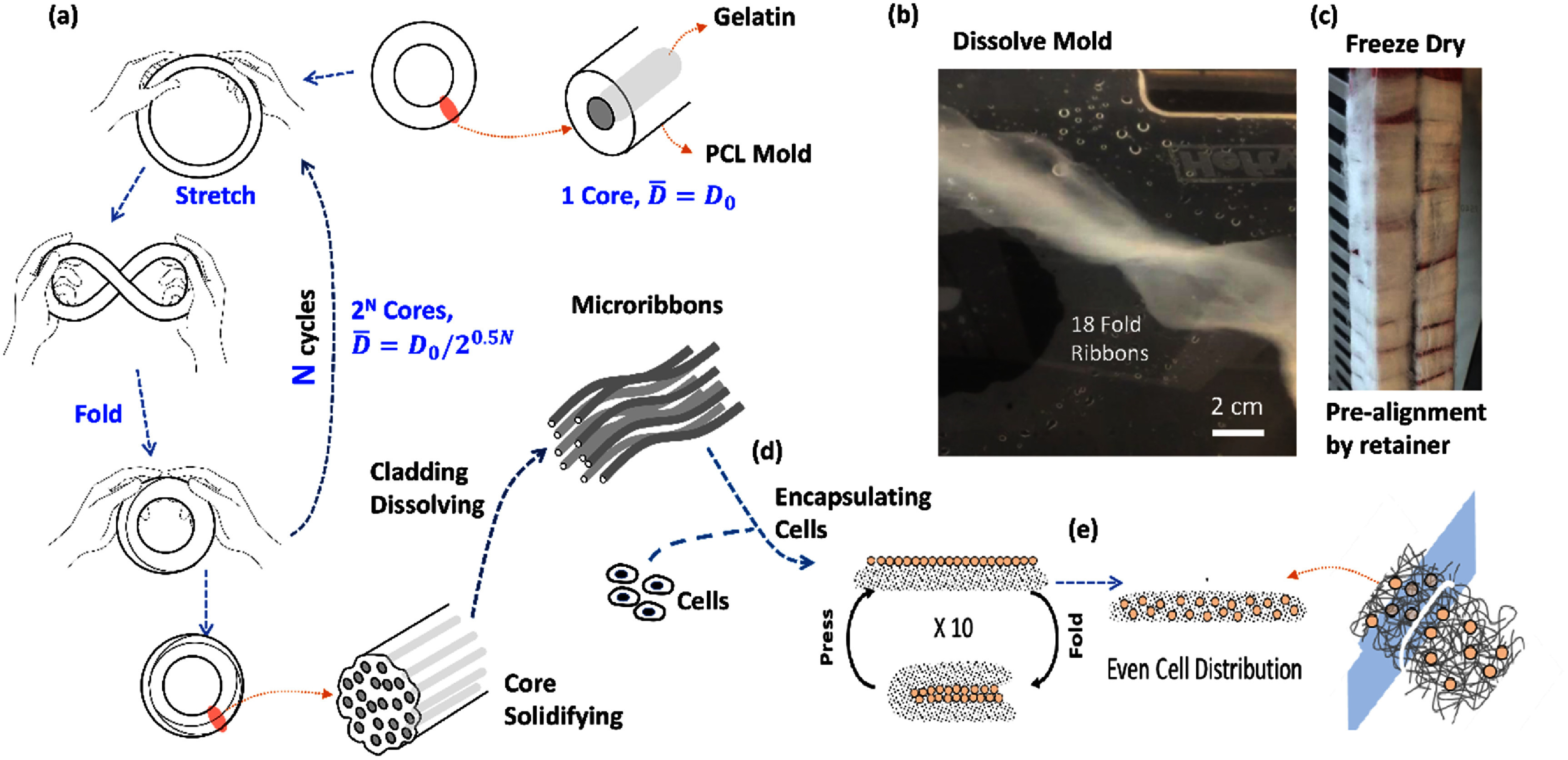
Preparation of microribbon-like building blocks and their use in forming 3D scaffolds with uniformly distributed cells. (a) Stretch-and-fold molding of gelatin core within a PCL mold to form microribbons of cellular or sub-cellular size. (b) PCL mold removal followed by chemical modification with methacrylic anhydride (MAA), acetic anhydride (AceA), or succinic anhydride (SucA). (c) Microribbons pre-aligned on retainers, freeze-dried, and stored at −20 °C. (d, e) Hydrated microribbons mixed with live cells formed a paste-like material that enabled uniform distribution. Upon crosslinking, a 3D scaffold was formed, where cell behavior was guided by microribbon alignment and geometry.

In this article, we implement this two-step method using gelatin, a denatured collagen. Type A porcine gelatin was formed into microscopic ribbon-shaped building blocks (microribbons) with widths on the order of a cell (∼10 *µ*m) via a novel procedure called **Dynamic Molding**, a fold-and-stretch process that sets ribbon size by the number of cycles (figure 1). We then added photocrosslinkable groups (methacrylate), thoroughly cleaned the ribbons, and performed a second crosslinking step to fuse the ribbons into a 3D network, yielding an interconnected, highly porous scaffold that distributes cells in 3D.

We next used post-functionalization to further tune the ribbons’ micromorphology, mechanics, swelling/shrinkage, degradation rate, and interfacial chemistry. Using gelatin’s native chemistry as a guide, we acylated primary amines (anhydride-mediated substitution) and varied the degree of methacrylation (GelMA) to control crosslink; we also incorporated and adjusted other functional groups to modulate hydrophilicity/hydrophobicity [6, 7]. We evaluated these modifications by quantifying indentation modulus, volumetric swelling, and degradation/stability of ribbons and scaffolds, and we assessed early attachment and proliferation of primary bovine meniscus-derived cells, a musculoskeletal model relevant to fibrous cartilage and tendon-like matrix formation.

Together, these experiments provided a first step showing that a two-step, building block-based hydrogel can be chemically designed using the same levers as traditional biopolymer hydrogels while providing porous architecture at the cell scale. This separation of hydrogel’s architecture and chemistry enabled independent control of pore size and material properties, establishing a new platform for targeted studies and future translational works.

## 2. Materials and methods

### 2.1. Synthesis and characterization of microribbons

**Microribbon Building Blocks Preparation**: Gelatin microribbons were prepared using a dynamic molding process that involved repetitive stretching (figure 1(a)), followed by chemical modification. A deformable mold was produced by melting polycaprolactone (PCL) pellets (Thermomorph, CAT#4336, MW 80 k–100 k g mol^−1^) and forming them into a hollow cylindrical rod with an inner diameter of 3 mm and an outer diameter of 15 mm. Type A porcine gelatin (Sigma-Aldrich, G2500) was fully dissolved in deionized water at a 1:1 weight ratio at 80–90 °C and injected into the PCL mold. The ends of the gelatin-filled PCL rod were sealed with additional melted PCL. For stretch-and-fold forming, the PCL mold was heated to 80 °C, above its melting point of approximately 60 °C, and shaped into a ring. The ring then underwent 18 cycles of stretching, twisting, and folding at 80 °C. Each cycle halved the cross-sectional area of the gelatin core and doubled the number of channels, while side compression during folding transformed the cores from cylindrical to ribbon-like shapes. After the prescribed number of cycles, the mold was cooled to room temperature to allow the gelatin to solidify by physical gelation. This process transformed the original round gelatin-filled core into a bundle of 262 144 (2^18) flat, micron-scale, gelatin-filled channels, that is, microribbons. Ribbon width was set by the number of fold-and-stretch cycles. Manual fabrication under a written standard operating procedure yielded batch-to-batch ribbon-width variation of no more than 25%, which did not measurably affect cellular responses in our prior experiments [6]. Ribbon length doubles with each fold-and-stretch cycle. For example, a 20 cm gelatin core would reach approximately 2.10 × 10^7 cm after 20 cycles (20 × 2^20 = 20 971 520 cm). In practice, such lengths were not maintained. After mold release, ribbons were cut in parallel into segments of about 1 ft (approximately 30 cm) for packaging, then trimmed to less than 1 cm for scaffold molding and 3D cell culture as needed. If required, the full extended length can be preserved by omitting this sizing step. The ribbons were extracted by dissolving and removing the PCL mold in a 9:1 volume ratio of acetone (Fisher Scientific, A949) and chloroform (Fisher Scientific, A412P). Gelatin microribbons remained intact during this step because they are insoluble in the selected solvent mixture.

**Modification by MAA:** Following microribbon fabrication, we performed methacrylation to identify an appropriate formulation for introducing methacrylate groups. The primary amine (NH₂) groups on lysine residues within the gelatin peptide serve as key reactive sites for chemical modification and are especially accessible to functional groups carrying an anhydride residue [8]. At a moderate pH (between 6 and 8), lysine reacts efficiently with anhydrides, forming stable amide bonds that covalently attach the functional groups to the gelatin backbone [9].

To introduce crosslinkable side chains, as-formed gelatin microribbons were suspended in methanol and reacted with varying amounts of methacrylic anhydride (MAA; Sigma-Aldrich, 276685) at 40 °C for 24 h. To estimate the required dosage, we assumed that approximately 4% of type A gelatin by weight consists of lysine, which reacts with MAA, based on the reported amino acid composition of gelatin derived from porcine skin collagen [10]. Based on this estimation, five different MAA concentrations were tested, corresponding to 1×, 2×, 3×, 4×, and 5× molar ratios of MAA to the total estimated moles of lysine in the microribbons. Samples were labeled accordingly (e.g. G-1XMAA for a 1:1 MAA-to-lysine ratio, G-2XMAA for 2:1, and so on, as listed in table 1). After reaction, the microribbons were thoroughly washed, dialyzed, lyophilized, and stored at −20 °C. Each treatment group was analyzed by FTIR (Fourier-transform infrared spectrometry) to evaluate methacrylation efficiency. The lowest MAA dosage that reached saturation of MAA-specific FTIR peaks (*n* times the estimated lysine molar amount) was selected for subsequent experiments.

**Table 1.** Initial chemical treatment of microribbon.

Sample No	Sample name	Methacrylate anhydride (molar ratio vs lysine)
1	G-1XMAA	1
2	G-2XMAA	2
3	G-3XMAA	3
4	G-4XMAA	4
5	G-5XMAA	5

**Figure 2.**
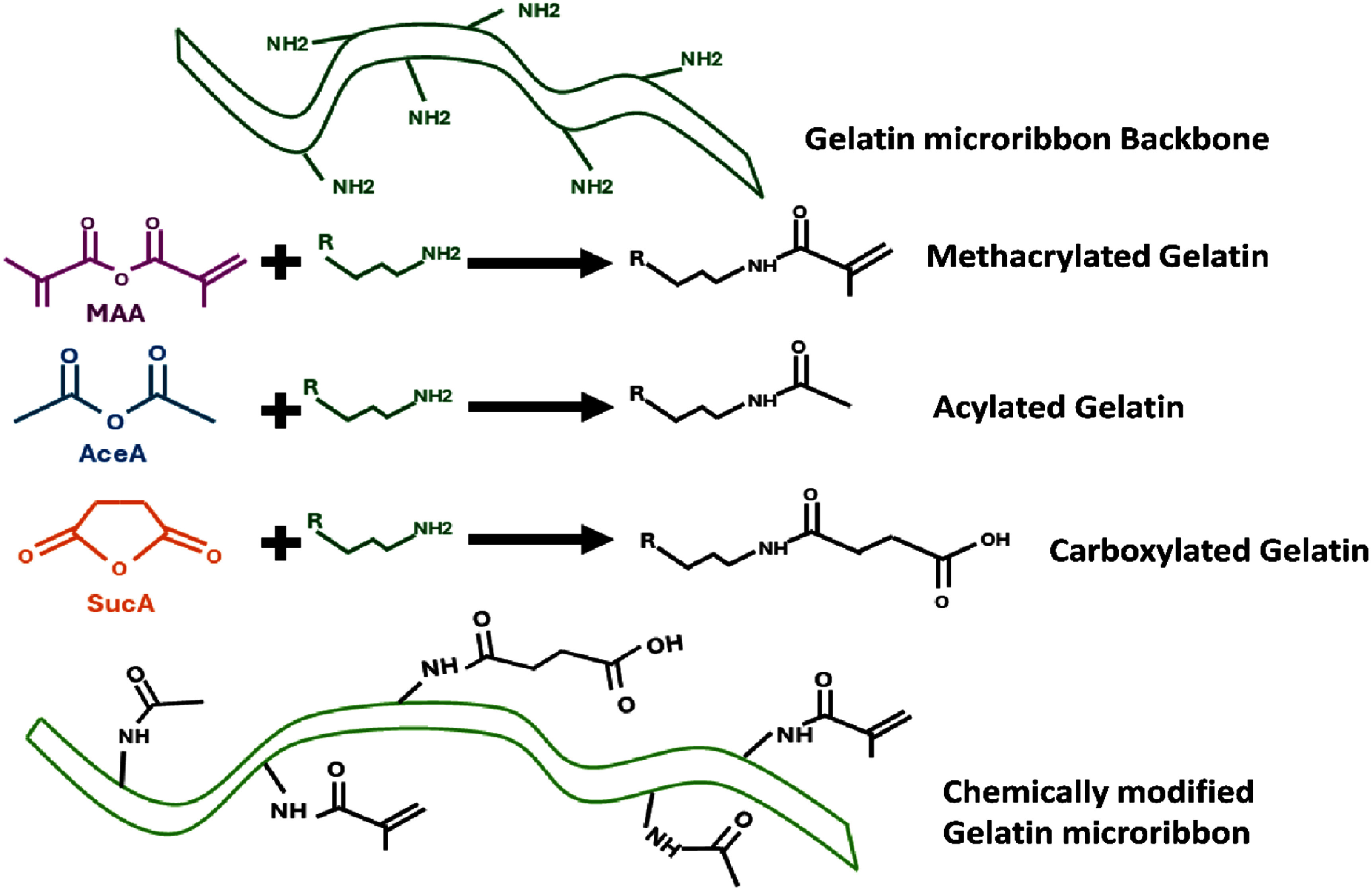
Chemical modification of gelatin microribbons. Primary amines on the gelatin backbone were functionalized with methacrylic anhydride (MAA), acetic anhydride (AceA), or succinic anhydride (SucA) to generate methacrylated, acylated, and carboxylated gelatin, respectively.

**Modification by MAA, –COOH, and –CH₃:** After determining the saturating MAA dosage, we next prepared ribbon-shaped building blocks with varying substitution levels of methacrylate (MAA), carboxylic (–COOH), and acetyl (–COCH₃) functional groups. Besides MAA, the additional groups were introduced using succinic anhydride (SucA; Sigma-Aldrich, 219552500) for –COOH groups and acetic anhydride (AceA; Sigma-Aldrich, 242845) for –COCH₃ groups (figure 2). We performed chemical modification after microribbon fabrication rather than before, since introducing methacrylate or anhydride groups prior to the stretch-and-fold molding process could interfere with gelatin chain interactions and disrupt consistent ribbon formation. All three reagents, i.e. MAA, SucA, and AceA, were added simultaneously at defined molar ratios relative to the estimated lysine content, as listed in table 2, and allowed to react for 24 h at 40 °C. Following reaction, the samples were purified and dried using the same procedures described above. The degree of lysine substitution by MAA, SucA, and AceA in each group was then analyzed using FTIR.

**Table 2.** Microribbon type based on chemical treatments.

Sample No	Sample name	Methacrylate anhydride (mol:mol)	Acetic anhydride (mol:mol)	Succinic acid (mol:mol)
1	G-n MAA	*n* ^a^	0	0
2	G-n MAA-AceA	*n*	1	0
3	G-n MAA-2AceA	*n*	2	0
4	G-n MAA-4AceA	*n*	4	0
5	G-n MAA-SucA	*n*	0	1
6	G-n MAA-2SucA	*n*	0	2
7	G-n MAA-4SucA	*n*	0	4

^a^ ‘*n*’ will be replaced by the lowest MAA dosage that reached saturation of MAA-specific FTIR peaks (n times the estimated lysine molar amount).

**ATR-FTIR:** ATR-FTIR spectra of the G-X MAA samples (gelatin microribbons with MAA modification) were recorded using a Bruker Invenio-R interferometer equipped with a platinum ATR accessory featuring a diamond crystal and a deuterated triglycine sulfate (DTGS) detector (Bruker Optics, Ettlingen, Germany). Spectra were collected from bundles of dried microribbon samples in the wavenumber range of 4000–400 cm^−1^, with a resolution of 4 cm^−1^. To quantify the saturation, point of methacrylation, integrated peak areas corresponding to methacrylate-specific wavenumber ranges were measured using Simpson’s Rule. These values were normalized and compared to non-methacrylated gelatin (G) as a reference to calculate the degree of modification (DoM), using a standard substitution formula. Microribbons without chemical modification were measured as control groups. DoM was determined by analyzing changes in the area under the amide II peak (∼1540 cm^−1^), using the following substitution-based formula [11],

\begin{align*}{\text{Degree of Modification }}\% { }\left( {{\text{DoM}}} \right) = \left( {\frac{{{\text{A Amide II}},{\text{ reference}} - {\text{A Amide II}},{\text{ sample}}}}{{{\text{A Amide II}},{\text{ reference}} - {\text{A Amide II}},{\text{ saturated}}}}} \right) \times 100\end{align*}

where A amide II, sample represents the integrated absorbance peak area of the amide II band (∼1540 cm^−1^) in modified samples, A amide II, reference corresponds to the peak area in unmodified gelatin (gelatin ribbons), and A amide II, saturated is the area from the microribbon with the lowest transmittance signal in amide II, indicating saturation with methacrylic anhydride (MAA). This method assumes amide II signal decreases proportionally to the number of NH₂ groups consumed during methacrylation, providing a reliable estimation of substitution efficiency [9, 10].

A second round of ATR-FTIR measurements was then conducted to evaluate the chemical modifications introduced by varying concentrations of AceA and SucA. The same amide II peak was used to assess the DoM, enabling direct comparison of chemical substitution efficiency across treatment groups. The DoM for these samples was also quantified using the same substitution-based formula (Formula 1), with un-modified gelatin ribbons as the reference (0% modification) and fully methacrylated gelatin (G-3MAA) as the saturated baseline (100% modification). Thus, the reported DoM values for AceA- or SucA-treated samples reflect the total cumulative modification resulting from both methacrylation and subsequent AceA or SucA reactions.

**Scanning electron microscope (SEM) analysis:** The morphology of the microribbons was examined using an environmental (SEM, FEI XL30). Prior to imaging, the samples were dried in desiccator overnight and then coated with a thin layer of platinum. The SEM was operated in high vacuum mode with a pressure of 1.5 torr. Imaging was conducted under an electron beam intensity of 1 kV. SEM images were acquired on lyophilized microribbons; therefore, the reported widths represent the dry state. To estimate their hydrated dimensions, we used the bulk **equilibrium swelling ratio (V_eq_)** of the corresponding hydrogels, defined as the ratio of swollen to dry volume (${V_{{\text{eq}}}} = \frac{{{V_{{\text{swollen}}}}}}{{{ }{V_{{\text{dry}}}}}}{ })$ (figure 5(c)). Assuming isotropic swelling, the linear dimensions of the microribbons increase with the cube root of the volumetric swelling ratio, i.e. hydrated width *=*dry width *× V*_eq_^1/3^ [1]. Under our experimental conditions (*V*_eq_ *≈* 1.0–1.4), this corresponds to approximately a 0%–12% increase in ribbon width upon hydration [12].

**Modulus of microribbons:** Given their extremely small size, the effective indentation modulus of microribbon, *E*_ind_, was measured using a Dimension Icon atomic force microscope (AFM, Bruker Nano) in 1X PBS, following establish protocol [13]. A microspherical colloidal tips (*R* ≈ 5 *µ*m, nominal *k* ≈ 0.03 N m^–1^, NanoAndMore, Arrow-TL1Au) was made by attaching the microsphere to the cantilever using M-Bond 610 adhesive glue (SPI). During the indentation, the probe tip was programmed to indent onto and compress the sample at a constant *z*-piezo displacement rate (10 *µ*m s^−1^) up to a maximum force of 40 nN. To ensure accuracy, indentations were performed in regions with surface roughness below 40 nm, identified by 5 *μ*m × 5 *μ*m contact mode surface scans. Each sample underwent indentation at a minimum of 10 different locations with three repeats at each location. Indentation force and depth were calculated from cantilever deflection and *z*-piezo displacement by calibrating deflection sensitivity on mica and determining the spring constant via thermal vibration. The tip-sample contact point was identified using a soft-material-specific algorithm, assuming no attractive forces [14]. The loading portion of each indentation curve was fitted to the elastic Hertz model using least-squares linear regression, allowing the calculation of the effective indentation modulus at the specified indentation rate using the formula:

\begin{align*}F = \frac{4}{3}{ }\frac{{{E_{{{ind}}}}{ }}}{{\left( {1 - {v^2}} \right){ }}}{R^{1/2}}{ }{D^{3/2{ }}}\end{align*}

where *E*_ind_ is the effective indentation modulus (Pascals), *F* is the indentation force (Newtons), *D* is the indentation depth (meters), *R* is the colloidal tip radius (meters), and *ν* is the microribbons Poisson’s ratio (*ν* = 0.46 for fully swollen hydrogels [15]). In this model, it is assumed that the polystyrene sphere has an infinite modulus (approximately 4 GPa) compared to that of the microribbons.

### 2.2. Fabrication and characterization of heterogeneously-crosslinked hydrogels

**Fabrication of microribbon hydrogels:** Microribbons were rehydrated in PBS containing 0.05% (v/v) of Lithium phenyl-2,4,6-trimethylbenzoylphosphinate (LAP; Sigma-Aldrich, 900889) as a photo-initiator and were incubated for 5 min. A cell strainer (VWR, 76327098) was then used to remove excess solutions from the microribbons. The microribbons were placed in a polypropylene, cylindrical mold that was 6 mm in diameter and 3 mm in thickness (figure 5(a)). The mold was positioned between two sterile transparent tapes and immediately exposed to blue light at a wavelength of 395 nm (6 cm distance, 2.5 mW cm^−2^ power) for 5 min to activate the crosslinking reaction (figure 5(b)). After exposure, the tapes were removed, and the hydrogel discs were placed in PBS for further use.

**Swelling of heterogeneously crosslinked** **hydrogels:** The swelling (or shrinking) behavior of the hydrogel samples was assessed gravimetrically by comparing the weights of the samples in their water-equilibrated state (*W*_w_) and their dry state (*W*_d_). Post crosslinking, each hydrogel disc was immersed in 1 ml of phosphate-buffered saline (PBS; J67802) at 37 °C for designated time points (0, 0.5, 1, 6, 12, 24, 48, and 96 h) until swelling equilibrium was reached. At each time point, excess surface PBS was gently blotted with absorbent paper, and the wet weight (*W*_w_) was recorded. The samples were then freeze-dried and weighed again to obtain the dry weight (*W*_d_) [12]. Swelling were calculated using the following formula, which accounts for the densities of both water and gelatin. The swelling percentage was determined by estimating the total volume change relative to the original mold volume:

\begin{align*}{\text{Swelling}}\,\left( \% \right) = \frac{{\left( {\frac{{{W_{\text{w}}} - {W_{\text{d}}}}}{{1.00}}} \right) + \left( {\frac{{{W_{\text{d}}}}}{{1.27}}} \right)}}{{{\text{Volume}}\,{\text{of}}\,{\text{mold}}}}\end{align*}

where *W*_w_ and *W*_d_ are the wet and dry weights of the sample in grams, respectively; 1.00 g cm^−3^ is the density of water; and 1.27 g cm^−3^ is the density of dry porcine gelatin (G1890, Sigma-Aldrich). The mold volume corresponds to the initial volume of the hydrogel at the time of fabrication and was used to normalize swelling across samples. All swelling values represent the mean of three independent replicates.

**Degradation of heterogeneously crosslinked** **hydrogels:** Disk-shaped hydrogels (6 mm in diameter and 3 mm in thickness) were prepared using microribbons with different functionalization formulas, with a consistent dry-to-wet weight ratios across all groups. Each hydrogel was incubated in 3 ml of either or PBS containing 2.5 mg ml^−1^ trypsin at 37 °C. At designated time points (1, 6, 12, 24, and 48 h, and 7, 14, and 21 d), the hydrogels were removed, gently blotted, freeze-dried, and weighed. The degradation ratio was calculated as the remaining dry weight (*W*_r_) divided by the initial dry weight (*W*_i_) [16]. All measurements were performed in triplicate,

\begin{align*}{\text{Degradation }}\left( {{\% }} \right) = \frac{{{\text{ Initial Weight of Sample}}\left( {{W_{\text{i}}}} \right) - {\text{Remaining weights of sample}}\left( {{W_{\text{r}}}} \right)}}{{{\text{Initial Weight of Sample}}\left( {{W_{\text{i}}}} \right)}}\end{align*}

***Compression test*:** Unconfined compression tests were conducted using a MARK-10 compression tester (Series 5, with digital force gauges, US code 9024100000) equipped with a 10 N load cell. The tests were performed in PBS solution at room temperature. Before testing, the diameter and thickness of the disc-shaped hydrogels were measured using digital calipers. A preload of approximately 2 mN was applied to ensure complete contact between the scaffold surface and the upper platen. To determine the compressive modulus, the upper platen was lowered at a rate of 10 mm min^−1^ until a maximum strain of 20% was achieved. Load and displacement data were recorded at a frequency of 10 Hz. Stress versus strain curves were plotted and analyzed within the 10%–20% strain range. Compression modulus was calculated from the initial linear slope of the stress–strain curve, to capture the elastic response of the material.

**Tensile test:** Tensile tests were conducted using Mark-10 system equipped with a pulling grip (Mark-10, G1062). Hydrogel samples cast in rectangular silicone molds with final dimensions of 18 mm in length (including a 12 mm gauge length, *L*_0_, and 3 mm at each end for gripping), 6 mm in width, and 3 mm in thickness. For this test, instead of being randomized, the ribbon-shaped building blocks were aligned parallel to the sample length and matched the loading direction. Before testing, the precise dimensions of each sample—length, width, and thickness—were measured using calipers, by the precision of 0.1 mm. Each sample was stretched at a constant rate of 10 mm min^−1^, corresponding to a nominal strain rate of 10 s^−1^, until reaching 20% strain. The strain was then held constant for 5 min to assess stress relaxation. Following relaxation, the samples were stretched to failure to determine ultimate tensile strength. Nominal stress (*σ*) was calculated by dividing the applied load by the undeformed cross-sectional area of each sample. Strain (*ϵ*) was calculated as the displacement of the grips divided by the initial gauge length (*L*_0_). The displacement of the grips was computed as follows:

\begin{align*}L = {L_0} + v \varepsilon\end{align*}

\begin{align*}\Delta L = L - {L_0}\end{align*}

Stress versus strain curves were generated, and the tensile modulus was calculated using the 10%–20% strain range. Yield stress was identified at the point where the tangent modulus $\frac{{{\text{d}}\sigma }}{{{\text{d}}\varepsilon }}$ exhibited the greatest rate of change with increasing strain for the elastic part of the stress–strain curve. To minimize water evaporation from the hydrogels, all measurements were conducted in a water bath enclosed in a transparent box at approximately 25 °C, unless otherwise specified.

### 2.3. Cell morphological change and proliferation in heterogeneously crosslinked hydrogels

**Culturing of bovine meniscus-derived cells:** Bovine knees from calves approximately 6 months old were obtained within 4 h post-mortem and dissected to expose the articular cartilage and menisci. Meniscus tissue was excised, minced into 1–3 mm fragments, and digested enzymatically to isolate meniscus-derived cells. The cells were cultured in tissue culture flasks containing Dulbecco’s modified Eagle medium (DMEM; Gibco, 11965092) supplemented with 10% fetal bovine serum (FBS; Invitrogen, 16140071) and 1% antibiotic-antimycotic solution (anti–anti; Gibco, 15 240 062). Cultures were maintained at 37 °C in a humidified atmosphere with 5% CO₂. Cells were passaged upon reaching 85%–90% confluence, and passage 2 cells were used for all subsequent experiments.

**Cell encapsulation and scaffolds preparation:** To fabricate cell-laden hydrogels, dried microribbon building blocks prepared with different functionalization formulas were rehydrated in culture medium (DMEM supplemented with 10% FBS and 1% anti–anti) at a 1:10 weight ratio and incubated for 30 min. The microribbons were then mixed with passage-2 bovine meniscus cells at a density of 2 million cells per cm^3^ and incubated for an additional 24 h to promote cell attachment. After incubation, the cell–microribbon mixtures were molded into cylindrical samples (6 mm diameter, 3 mm thickness) and photo-crosslinked into solid scaffolds using light (395 nm, 2.5 mW cm^−2^) for 5 min.

**Fluorescent staining of actin cytoskeleton and** **cell nuclei:** On days 1, 7, and 14 after hydrogel preparation, samples were fixed in 4% paraformaldehyde (PFA; 158127) in PBS for 30 min at room temperature, then permeabilized with 0.05% Triton X-100 (Thermo Fisher, T8787) in PBS for 5 min. Fluorescent staining was performed using phalloidin (ab176756) to label the actin cytoskeleton and Hoechst 33 238 (Invitrogen, H21486) to stain cell nuclei, following standard protocols. 3D fluorescence images were captured using a confocal fluorescence microscope (LSM700, Carl Zeiss, Oberkochen, Germany).

### 2.4. Statistical analysis

Data are presented as mean ± SD, with *n* denoting independent biological replicates. For comparisons involving a single factor (e.g. mechanical properties across formulations at one time point), statistical differences were assessed using one-way ANOVA followed by Tukey’s HSD post-hoc test (*α* = 0.05). For experiments involving two independent variables (e.g. *sample type* and *time*), data were analyzed using a two-way ANOVA including the interaction term, followed by Tukey-adjusted simple-effects comparisons when appropriate. Groups that do not share a letter are significantly different (*p* < 0.05). Statistical significance was defined as *p* < 0.05 for all tests.

## 3. Results and discussion

### 3.1. Methacrylation of gelatin microribbons, which enables heterogeneous crosslinking

**Figure 3.**
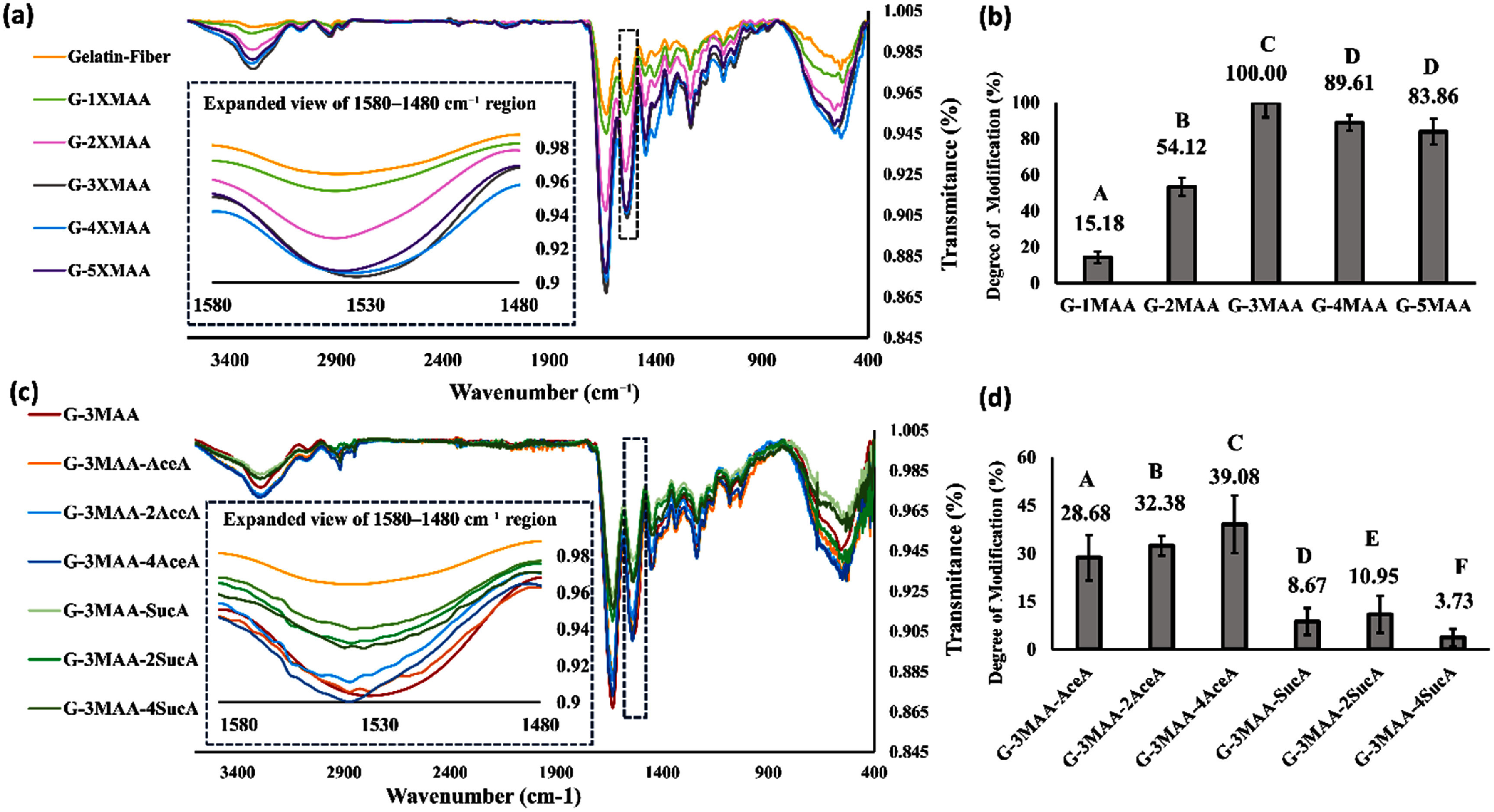
(a) FTIR spectra of unmodified microribbons and microribbons modified with increasing concentrations of methacrylic anhydride (G-1MAA to G-5MAA). The amide II band (∼1540 cm^−1^, N–H bending and C–N stretching) decreased with higher methacrylation. A zoomed-in view (1580–1480 cm^−1^) highlights this trend. (b) Degree of methacrylation (DoM) calculated from FTIR peak intensities increased with MAA dosage but plateaued beyond 3×. (c) Additional treatment with AceA or SucA altered the substitution efficiency relative to MAA alone. (d) SucA had a stronger effect than AceA in reducing modification efficiency. Data are presented as mean ± SD (*n* = 3). Statistical significance in (b, d) was determined using one-way ANOVA with Tukey’s HSD post hoc test (*p* < 0.05). Groups that do not share a letter are significantly different (*p* < 0.05).

In the first stage of the experiments, we determined the MAA dosage required to saturate substitution of lysine side chains in gelatin. After fabricating microribbon-like building blocks using the dynamic molding method, the microribbons were treated with methacrylic anhydride (MAA) at five molar ratios: 1, 2, 3, 4, and 5 times the estimated lysine content in gelatin. Fourier transform infrared spectroscopy (FTIR) was used to confirm chemical modification and assess the extent of methacrylation at each concentration (figure 3(a)).

In the FTIR spectra (figures 3(a) and (c)), three peaks (1635, 1540, and 920 cm^−1^) were identified as indicators of methacrylate bonding to gelatin, each potentially usable for quantifying methacrylation. Among them the 1540 cm^−1^ peak was selected. The 1635 cm^−1^ peak corresponds to C=C stretching of methacrylate but overlaps with the amide I band from both native gelatin peptides and the new amide bond formed between MAA and lysine (C=O bonds, ∼1635–1650 cm^−1^), making it sub-optimal for quantification [17]. The 920 cm^−1^ peak, corresponding to the C–H out-of-plane bending of methacrylate C=C bonds, showed weaker signals and smaller differences between groups [18]. In contrast, the 1540 cm^−1^ peak, associated with N–H bending and C–N stretching in the amide II band, provided a clearer and more quantifiable marker of methacrylation [19].

Microribbons with MAA dosage of 3× (G-3MAA), which showed the lowest Amide II transmittance signal, was identified as the saturated sample and used in Formula 1 to calculate DoM (figure 3(a)). As the MAA dosage increased from 1× to 3× the estimated molar amount of lysine in gelatin, all methacrylate-associated peaks decreased (figure 3(a)), calculated as described in the methods section, indicating a higher level of lysine substitution. Results showed a direct correlation between increasing methacrylic anhydride (MAA) concentrations and DoM, with DoM values rising from 15.18% to 100% as the MAA dosage increased from 1 to 3. This suggests a continuous increase in methacrylation with an increasing MAA input. However, further increasing the MAA dosage to 4× and 5× resulted in minimal additional change, indicating that a 3:1 MAA-to-lysine ratio approaches saturation for MAA modification [20]. This 3× dosage was then selected as the standard condition for microribbon-like building block preparation. Beyond this concentration, additional MAA did not further decrease the Amide II signal, suggesting that the majority of available amine groups were already consumed at the 3× MAA dosage. For this reason, dosages above 3× MAA were not pursued in the subsequent experiments involving the addition of AceA and SucA.

### 3.2. Modifying microribbons with carboxylic and acetyl groups

Having decided on the standard treatment dosage of MAA, which introduced crosslinkable side chains to the microribbon-shaped building blocks, we next examined the incorporation of other function groups known to influence the physical properties and cell behavior of the resulting hydrogels. These included succinic anhydride (SucA) and acetic anhydride (AceA), which introduce carboxylic groups (–COOH) and acetyl groups (–COCH₃), respectively.

In this stage of the experiment, gelatin microribbons fabricated by dynamic molding were simultaneously treated with a fixed dosage of MAA (3× molar ratio relative to lysine) and varying dosages of SucA and AceA, as listed in table 2. FTIR analysis of microribbons from different treatment groups revealed characteristic peaks at consistent positions, with peak intensities varying across groups.

Following the same approach used in the MAA-only modification, we quantified overall lysine substitution by the changes in the 1540 cm^−1^ peak, which corresponds to new amide bond formation. It is important to note that this peak reflects amide bonds formed by not only MAA but also by AceA and SucA, which compete with MAA for lysine residues [21]. This competition was evidenced by a decrease in the ∼1540 cm^−1^ peak, corresponding to the methacrylate C=C bond, as the dosage of SucA or AceA increased (figure 3(c)).

The introduction of competing groups reduced not only MAA substitution but also the overall degree of lysine modification. Specifically, increasing the concentration of succinic anhydride (SucA), which introduces carboxylic acid (–COOH) groups, resulted in a marked decrease in total substitution. The DoM decreased from approximately 100% to 3.73% as the SucA molar ratio increased from 0 (representing G-MAA at saturation level) to 4× the estimated lysine content. This reduction was likely due to changes in the reaction environment, particularly the drop in pH caused by the formation of succinic acid as a byproduct of SucA reacting with primary amines. In contrast, increasing acetic anhydride (AceA) dosage led to only a modest decrease in total lysine substitution [22].

To isolate the effects of functional group identity, all reactions in this study were performed in unbuffered aqueous solution at an initial pH of 7. This condition was chosen as a baseline to minimize external influences from buffering agents and allow direct comparison across modification groups. For applications requiring tighter control of substitution efficiency, buffering agents such as disodium phosphate (Na₂HPO₄) may be used to help stabilize pH during the reaction and mitigate pH-dependent variability [20].

### 3.3. Modification by carboxylic and acetyl groups may enhance or disrupt microribbon’s structural integrity

SEM images of dialysis-treated and lyophilized microribbon-shaped building blocks (referred to as ‘microribbons’ hereafter) were used to evaluate ribbon morphology and measure ribbon width across samples with different chemical treatments.

The treatments with MAA, AceA, and SucA, along with the subsequent dialysis and freeze-drying steps, were performed while preserving microribbon alignment using a retainer (figure 1(c)), which kept the microribbons’ originally aligned state. Preserving this alignment during hydrogel formation results in channel-like internal pores that support cell alignment and the formation of linear tissues such as tendons and muscles. Alternatively, the alignment can be disrupted to produce more randomized structures when isotropic tissue formation is preferred [14].

**Figure 4.**
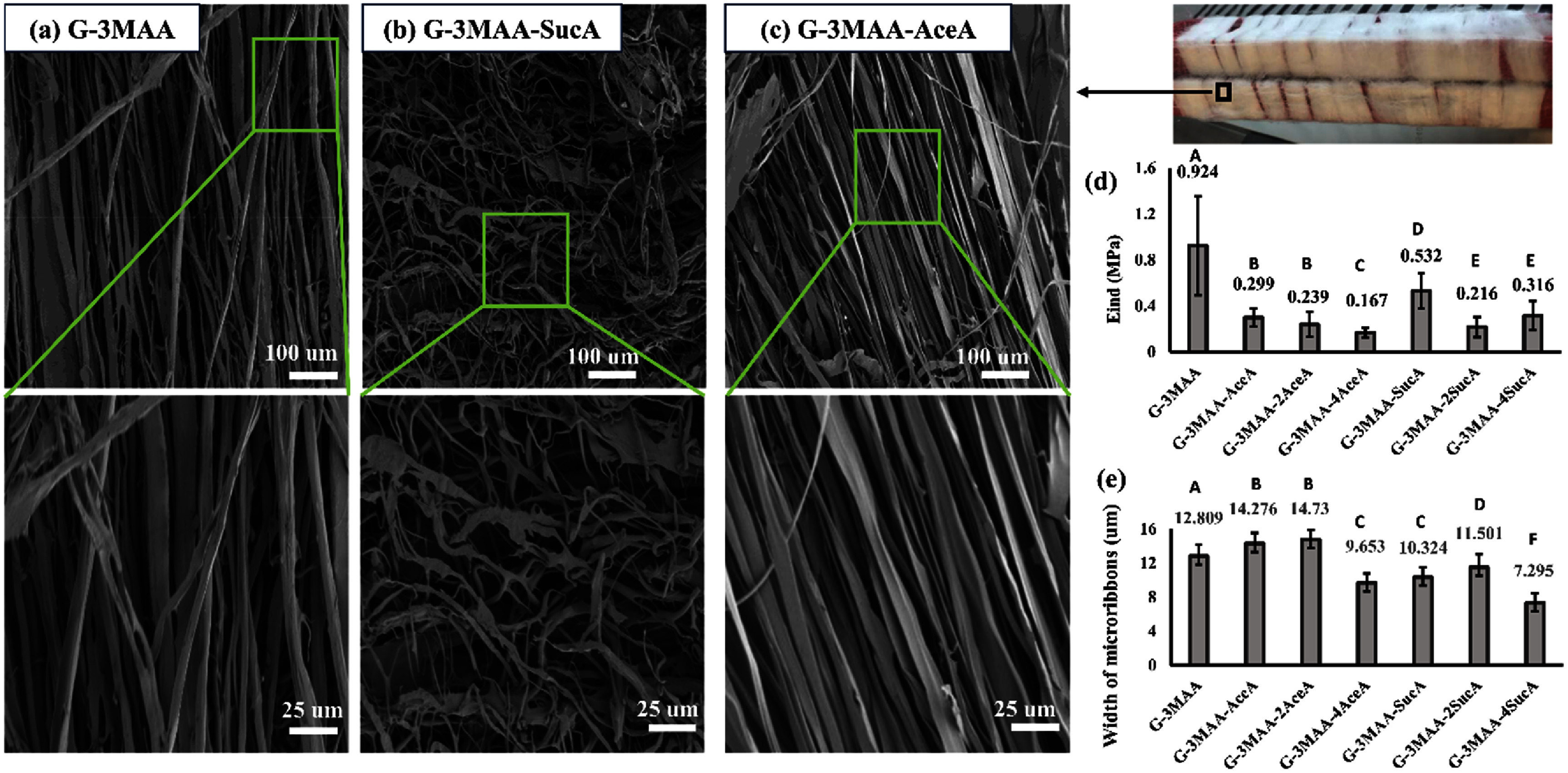
Structural and microscopic mechanical characterization of chemically modified microribbons. (a)–(c) SEM images showing microribbon morphology and alignment for G-3MAA, G-3MAA-SucA, and G-3MAA-AceA, with zoomed-in views highlighting differences in architecture. G-3MAA and G-3MAA-AceA maintained alignment, while G-3MAA-SucA displayed disrupted structures and branching. (d) Indentation modulus (*E*_ind_) of single microribbons measured by AFM indentation. Additional chemical modifications reduced stiffness from ∼0.9 MPa to ∼0.15 MPa. (e) Microribbon width after AceA or SucA treatment, with AceA increasing width and SucA reducing it. Error bars indicate standard deviation (*n* = 3). Statistical analysis in (d, e) was performed using one-way ANOVA followed by Tukey’s HSD post-hoc test (*α* = 0.05). Groups that do not share a letter are significantly different (*p* < 0.05).

SEM images showed consistent microribbon alignment across different treatment groups (figure 4(a)). Alignment was well maintained in microribbons treated with MAA alone (G-3MAA, gelatin microribbons modified with 3 molar equivalents of MAA) and in those treated with both MAA and AceA (e.g. G-3MAA-AceA = 3 molar MAA + 1 molar AceA; G-3MAA-2AceA = 3 molar MAA + 2 molar AceA; G-3MAA-4AceA = 3 molar MAA + 4 molar AceA), which introduced hydrophobic CH₃ groups. This structural stability is likely due to the limited impact of hydrophobic substitution on gelatin’s solubility in water at room temperature, where unmodified gelatin microribbons retain physical gelation. As a result, microribbon architecture was preserved during extended aqueous exposure throughout dialysis and freeze-drying [23].

In contrast, microribbons treated with SucA, which introduces COOH groups, showed visible disruption despite the confinement by the retainer. This can be attributed to the carboxylic groups lowering pH and forming ionic interactions with native functional groups in the gelatin matrix, possibly disturbing the internal microstructure [24]. In addition, new carboxylic groups may have increased gelatin solubility, reducing the microribbon stability during dialysis [25].

SEM-based measurements showed that the average width of microribbons treated with MAA alone was 12.81 *µ*m, serving as the baseline. The addition of AceA caused minimal changes, with widths of 14.28 *µ*m and 14.73 *µ*m for G-3MAA-AceA and G-3MAA-2AceA, respectively. However, excessive AceA substitution in G-3MAA-4AceA led to a significant reduction in width to 9.65 *µ*m, likely due to disruption of the gelatin matrix and reduced structural integrity.

Although SucA treatment disrupted ribbon alignment, lower concentrations had minimal effect on ribbon width, with measurements of 10.32 *µ*m and 11.50 *µ*m for G-3MAA-SucA and G-3MAA-2SucA, respectively. The slight reduction in width may be attributed to increased solubility and minor material loss during dialysis. At higher SucA concentrations, the average ribbon width decreased more substantially to 7.30 *µ*m, likely due to further enhanced solubility and greater material loss during processing [26].

### 3.4. Carboxylic and acetyl group modifications reduce microribbon modulus but increase mechanical consistency

The modulus of microribbons may directly influence adherent cells through the mechano-sensing signaling pathway described earlier [27]. Similarly, the consistency of modulus along the ribbon surface may also affect cell behavior. To assess these parameters, AFM analysis was performed on rehydrated microribbons to measure both their modulus and spatial consistency, reported as compression effective modulus *E*_ind_ in megapascals (MPa). Measurements were conducted on uncrosslinked microribbons, with methacrylate groups (MAA) remaining unreacted. Nano-indentation data were used to evaluate the effects of different chemical treatments.

The AFM results showed that among all groups, the G-3MAA groups, representing microribbons treated with MAA only, exhibited the highest modulus. Notably, this group also displayed the largest mechanical heterogeneity, indicated by the largest standard deviation in compression modules (figure 4(e)).

The modulus decreased when carboxylation (SucA) and acetylation (AceA) molecules were introduced in addition to MAA. This may be due to the increased variety of chemical groups in the gelatin matrix, which could disrupt the gelatin matrix and reduce structural integrity, as also suggested from the SEM data (figure 4(a)).

Despite reducing modulus, acetylation by AceA improved mechanical consistency. This may be attributed to the hydrophobic nature of AceA, which likely reduced water solubility and helped preserve microribbon structure during dialysis. In contrast, carboxylation by SucA significantly increased mechanical heterogeneity compared to AceA-treated groups [28, 29]. The greater variation in compression modulus suggests that the hydrophilic carboxylic groups introduced by SucA enhanced water uptake and erosion during dialysis, leading to increased non-uniformity in microribbon structure, as also evident in the SEM images (figure 4(a)).

### 3.5. Carboxylation enhances swelling of heterogeneously crosslinked hydrogels, while acetylation enhances shrinkage of them

The kinetics of water retention in hydrogels, often assessed through the hydrogel’s swelling behavior, play a critical role in cell survival by influencing nutrient diffusion, waste removal, and the maintenance of a hydrated environment necessary for cellular function. In medical applications, the swelling and shrinkage of hydrogel implants directly impact their ability to remain in place within tissue defects and to integrate with host tissue over time. To evaluate swelling in microribbon-based, heterogeneously crosslinked hydrogels, disk-shaped samples (6 mm diameter × 3 mm thickness) were prepared from different microribbon formulations and incubated in PBS at 37 °C for defined time intervals, as detailed in the Methods section.

**Figure 5.**
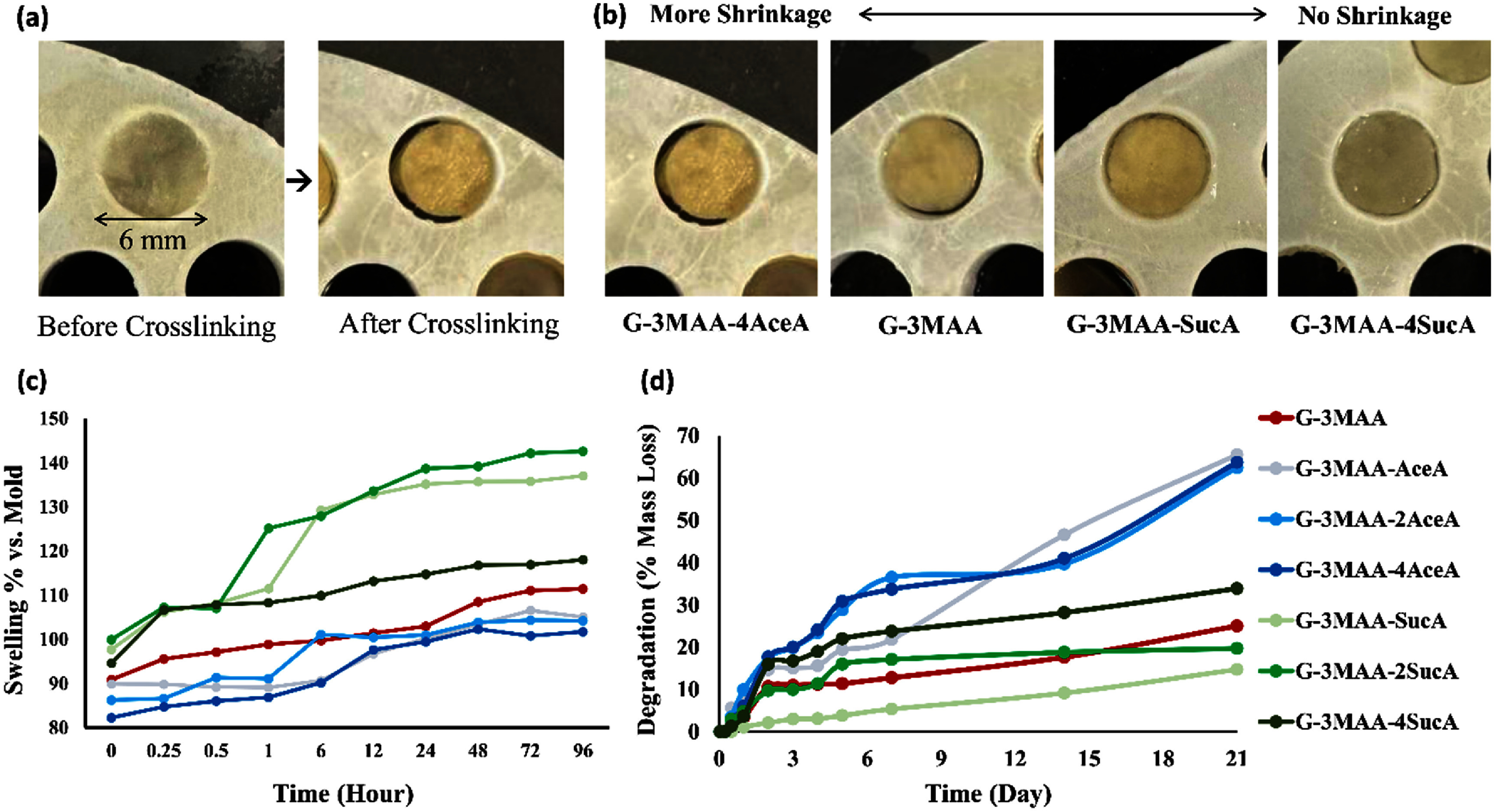
Swelling and degradation behavior of heterogeneous hydrogels formed by crosslinking microribbon-like building blocks pre-treated with different chemical formulations. (a) Hydrogels before and after crosslinking in the mold. (b) Volume shrinkage immediately after crosslinking. (c) Swelling behavior during 96 h incubation in PBS, with SucA-treated groups showing the highest equilibrium swelling. (d) Degradation profiles over 21 d in PBS at 37 °C, where AceA-treated groups degraded significantly faster than the MAA-only and SucA-treated groups. G-3MAA-4AceA exhibited the greatest mass loss (>60%). Color-coded labels correspond to the curves shown in (c) and (d).

As shown in figure 5(b), all samples exhibited volume shrinkage after crosslinking, indicated by swelling ratios below 1 relative to the original mold volume (figure 5(c)). Microribbon crosslinking led to initial shrinkage, likely due to the contraction caused by the crosslinking between the MAA groups within each microribbon and between adjacent microribbons [29]. In contrast, increased carboxylation enhanced swelling, counteracting the contraction by MAA crosslinking, and helped preserve the original volume, resulting in volume ratios closer to 1. Hydrogels made from AceA-treated microribbons exhibited the greatest shrinkage, likely due to the water-repellent effect of the hydrophobic –CH₃ groups introduced by AceA [30].

After incubation in PBS at 37 °C, all samples exhibited volumetric swelling over time relative to their post-crosslinking size (figure 5(c)). Hydrogels formed from SucA-treated microribbons, which contained more hydrophilic functional groups, showed significantly higher swelling rate compared to those modified with MAA alone. In particular, hydrogels from the G-3MAA-2SucA group expanded from a volume ratio of ∼1.0 to over 1.4 relative to the original mold. In contrast, hydrogels prepared from MAA-only microribbons, which initially shrank slightly after crosslinking (∼0.9 volume ratio), swelled moderately to ∼1.1 ratio after 96 h. Hydrogels made from AceA-treated microribbons, which carried more hydrophobic groups, exhibited minimal net volume change and swelled only to match the original mold volume.

Increasing the dosage of SucA, which introduces negatively charged carboxylic groups and enhances water affinity, led to an increased swelling rate. This trend was evident when the SucA dosage was raised from 1× to 2× the estimated lysine content (G-3MAA-1SucA and G-3MAA-2SucA). Interestingly, the group with the highest SucA dosage (G-3MAA-4SucA) exhibited a significantly lower swelling ratio over time compared to the lower-dose groups. The mechanism behind this unexpected result remains unclear. One possible explanation is that excessive SucA competed with MAA for lysine residues and altered the reaction pH, thereby reducing the overall introduction of MAA groups, as discussed in the FTIR analysis (figure 3(b)). This may have decreased the crosslinking density within individual microribbons and compromised their structural stability in water. As a result, the G-3MAA-4SucA group may have undergone partial dissolution during incubation, leading to volume loss that appeared as a lower swelling ratio.

### 3.6. Heterogeneously crosslinked hydrogel’s degradation is enhanced by acetylation and suppressed by carboxylation

The degradation behavior of the heterogeneously crosslinked hydrogels, with or without trypsin, was evaluated by incubation in PBS at 37 °C for up to 21 d (figure 5(d)). In the presence of trypsin, all samples degraded rapidly. Complete degradation within 12 h was observed for G-3MAA-AceA, G-3MAA-2AceA, G-3MAA-4AceA, and G-3MAA-4SucA. Hydrogels made from microribbons treated with MAA alone (G-3MAA) degraded more slowly, showing 70% weight loss after 1 d and complete degradation by day 3. G-3MAA-SucA and G-3MAA-2SucA exhibited slower initial degradation, with 57% and 40% weight loss after 1 d, respectively. These groups continued degrading gradually, with 87.8% and 86.0% weight loss by day 7, and full degradation reached by day 10.

In PBS without trypsin, all groups degraded significantly more slowly. Samples treated with MAA alone (G-3MAA) and those containing lower amounts of COOH groups (G-3MAA-SucA and G-3MAA-2SucA) showed 25%, 14.7%, and 19.6% weight loss, respectively, after 21 d. Samples containing AceA, which introduces hydrophobic CH₃ groups, exhibited faster degradation during the first 7 d compared to MAA-only samples (figure 5(d)). Although increasing AceA concentration accelerated early degradation, all AceA-containing groups reached similar levels of degradation by day 21. In contrast, samples treated with SucA, which increases hydrophilicity, showed a continuous increase in degradation throughout the entire 21 d period.

Overall, samples modified with AceA degraded the fastest, followed by MAA-only samples, and finally those containing SucA. The rapid degradation in AceA groups may be attributed to reduced crosslinking density of MAA, due to lower MAA content, which likely increased hydrolysis rate and enzymatic accessibility [31].

It remains unclear why SucA-containing heterogeneous hydrogels were more stable in PBS than the MAA-only group, despite exhibiting greater swelling and water uptake—factors typically associated with accelerated hydrolysis. One possible explanation is that the carboxylic groups introduced by SucA may form ionic interactions with oppositely charged residues within the matrix, such as arginine, histidine, and unmodified lysine, thereby enhancing structural stability [32].

### 3.7. Compressive strength of hydrogels is enhanced by carboxylation but not acetylation, while both modifications lower tensile strength

**Figure 6.**
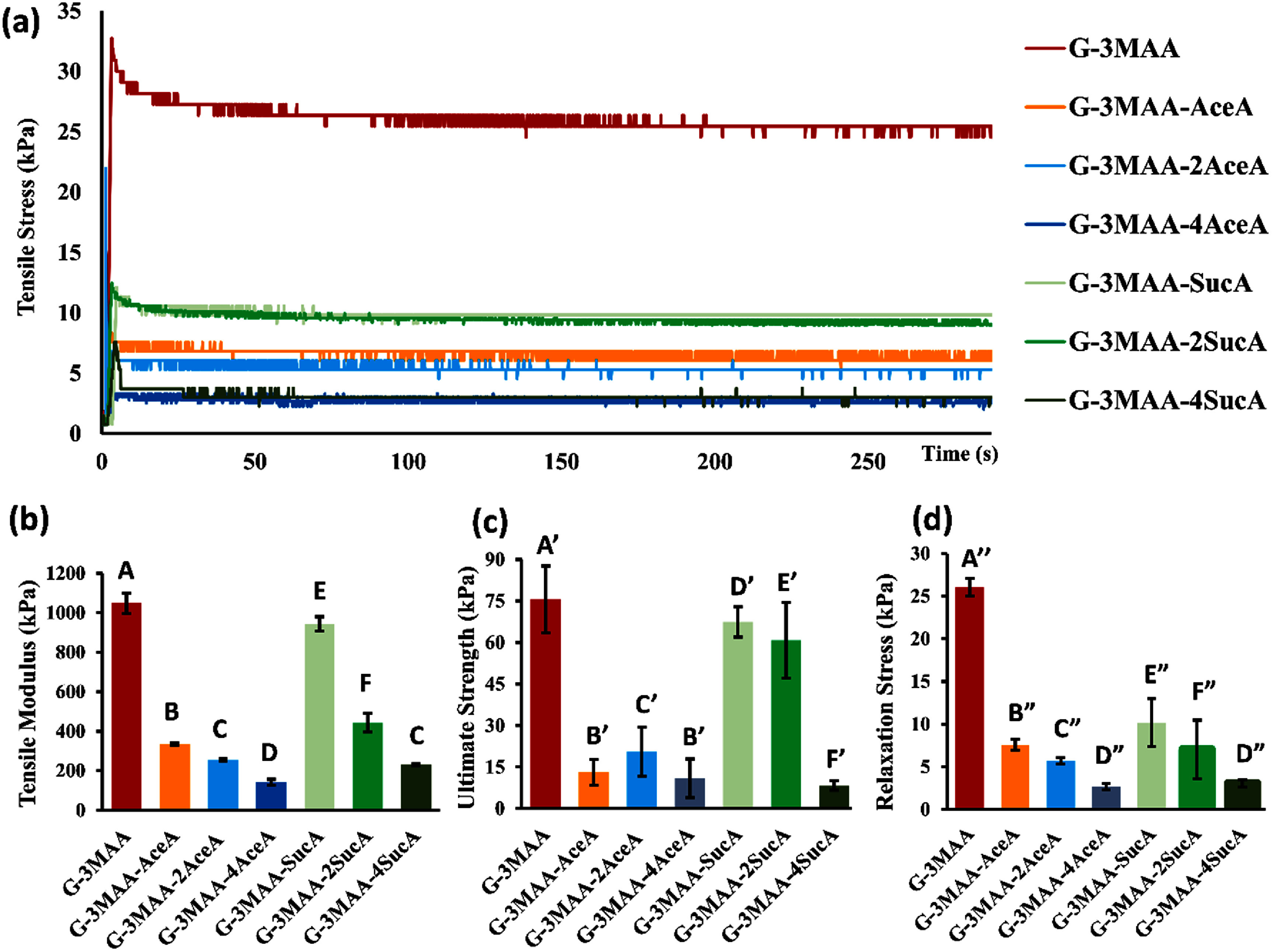
Bulk unconfined tensile test of heterogeneous hydrogels formed by crosslinking microribbon-like building blocks pre-treated with different chemical formulations. (a) Stress–relaxation curves (20% strain) for groups treated with MAA only (G-3MAA) or MAA plus AceA or SucA at varying levels (G-3MAA-AceA, −2AceA, −4AceA; G-3MAA-SucA, −2SucA, −4SucA). (b) Tensile modulus from the 10%–20% strain region of the stress–strain curves. (c) Ultimate tensile strength at failure. (d) Relaxation stress measured after 5 min at 20% strain. Compared to the MAA-only group (G-3MAA), both AceA and SucA treatments reduced tensile modulus, strength, and relaxation stress, with the largest decreases observed in the AceA groups. Error bars indicate standard deviation (*n* = 3). Statistical differences in (b)–(d) were analyzed using one-way ANOVA followed by Tukey’s HSD post-hoc test (*α* = 0.05). Groups that do not share a letter are significantly different (*p* < 0.05).

In contrast to the AFM-based measurements performed on individual microribbons, macroscopic tensile and compression tests were conducted on hydrogels formed from microribbons. Prior to testing, heterogeneously crosslinked hydrogels were incubated in PBS for 96 h to allow them to reach swelling equilibrium. During mechanical testing, stress–time and stress–strain curves were recorded (figure 6(a)), from which tensile modulus, compressive modulus, relaxation stress, and ultimate tensile strength were calculated.

Tensile testing showed that treatment of microribbons with SucA or AceA made the resulting hydrogels less resistant to extension, as indicated by reductions in tensile modulus and ultimate tensile strength (figures 6(b) and (c)). Increasing the dosage of either group further reduced these mechanical properties. A similar trend was observed in relaxation tests, where AceA- and SucA-treated samples exhibited lower equilibrium tensile moduli (figure 6(d)).

Between the two groups, AceA consistently caused a greater reduction in tensile properties than SucA [33]. For example, compared to the MAA-only group (G-3MAA), the tensile modulus dropped by about 10% in G-3MAA-SucA and by 68% in G-3MAA-AceA. This more pronounced weakening is likely due to a greater reduction in crosslinking density caused by the competition between AceA and MAA for lysine substitution [33]. In contrast, microribbons treated with SucA maintained higher modulus, possibly due to ionic attraction between the introduced carboxylic groups and other functional groups in the matrix, which may help stabilize the hydrogel network [34].

**Figure 7.**
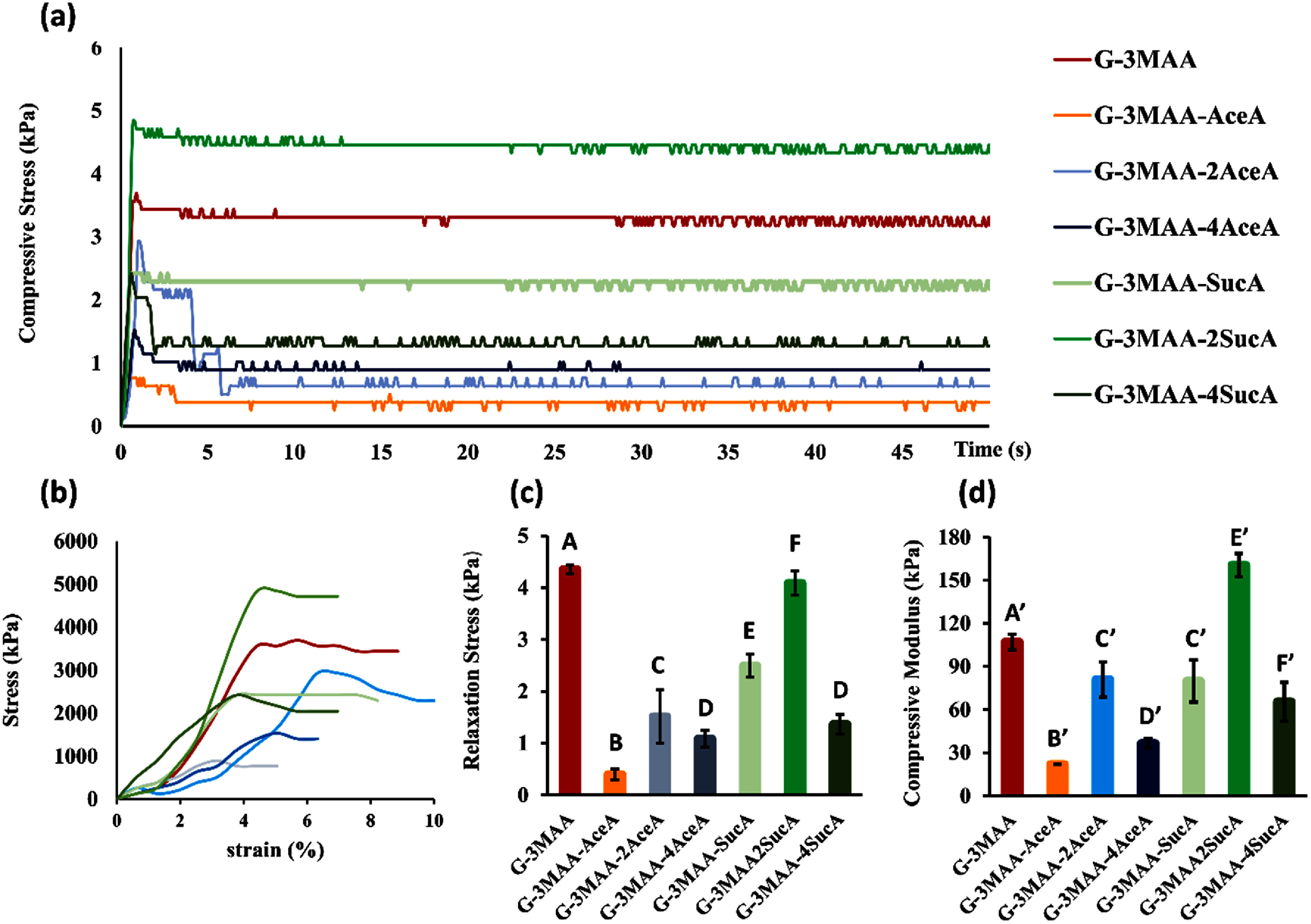
Bulk unconfined compression analysis of heterogeneous hydrogels made by crosslinking the microribbon-like building blocks that were pre-treated with different chemical formulations. ((a) Stress–relaxation curves (20% strain, 5 min) (b) Stress–strain curves to 10% strain. (c) Compressive modulus calculated from the 10%–20% linear region of the stress–strain curves. (d) Relaxation stress measured from the plateau phase (5 min). G-3MAA showed the highest compressive stiffness and stress retention. Both AceA and SucA treatments reduced these values in a dose-dependent manner, with AceA having a stronger softening effect. Error bars indicate standard deviation (*n* = 3). Statistical differences in (c, d) were determined using one-way ANOVA followed by Tukey’s HSD post-hoc test (*α* = 0.05). Letters above bars denote Tukey groupings within each subplot; identical letters (*A, A*′) indicate independent statistical analyses for panels (c) and (d), respectively. Groups that do not share a letter are significantly different (*p* < 0.05).

Interestingly, SucA had a different effect on compression compared to tension. Hydrogels formed from microribbons treated with a moderate dosage of SucA (G-3MAA-2SucA) exhibited the highest compressive modulus (both instantaneous and equilibrium) exceeding that of the MAA-only group (figures 7(a), (b), and (d)). In contrast, either increasing or decreasing the SucA dosage from this level reduced the compressive modulus below that of G-3MAA. This complex result can be explained by the combined effects of SucA on MAA substitution, crosslinking density, and swelling behavior. At the intermediate SucA level, the swelling pressure generated by internal carboxylic charges may have been strong enough to resist compression and offset the weakening caused by reduced MAA bonding and lower MAA crosslinking density due to competition from SucA [22, 35]. In contrast, lower SucA levels provided insufficient swelling pressure, while excessive substitution with SucA significantly reduced MAA content and crosslinking, weakening the hydrogel [36].

Unlike the SucA-treated samples, increasing the AceA dosage consistently reduced compressive modulus, both instantaneous and equilibrium. This trend is attributed to the presence of CH₃ groups from AceA, which do not contribute to swelling pressure but still compete with MAA for lysine residues, thereby reducing crosslinking density [35].

The compressive results changed by chemical modifications show that the mechanical properties of heterogeneously crosslinked hydrogels can be tuned by balancing crosslinking groups (MAA), hydrophobic groups (AceA), and hydrophilic groups (SucA). The dosage of each modification can be optimized based on the needed cell behavior and the specific tissue engineering application.

### 3.8. Carboxylation enhances cell proliferation, while acetylation reduces it

**Figure 8.**
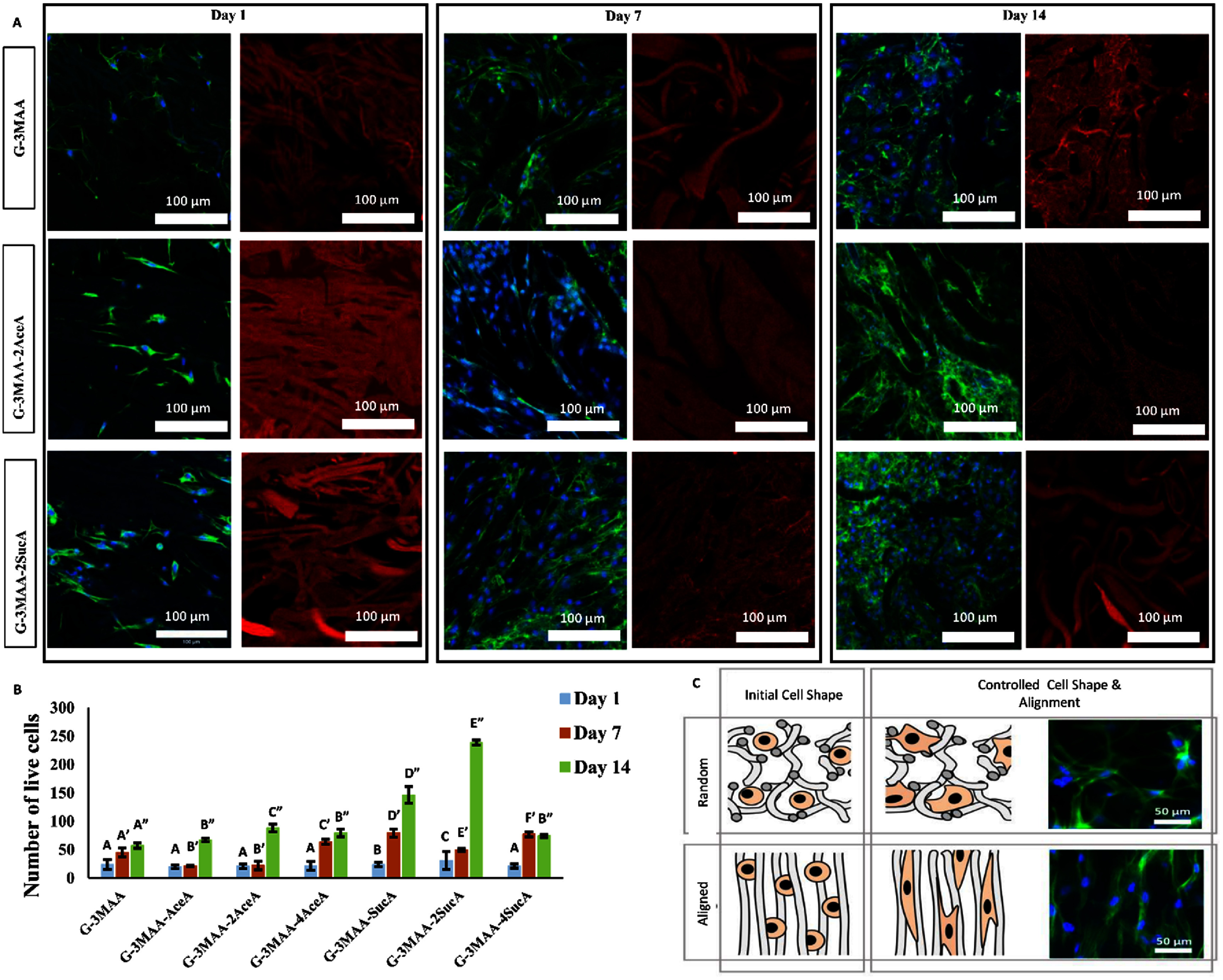
Day 1, 7, and 14 confocal microscopy images of cell-laden heterogeneous hydrogels formed by crosslinking microribbon-like building blocks pre-treated with different chemical formulations (G-3MAA, G-3MAA-2AceA, and G-3MAA-2SucA). (a) Confocal images showing nuclei (blue), F-actin (green), and microribbons (red). (b) Cell number in each scaffold over time (days 1, 7, and 14). (c) Microribbon alignment further influences 3D cell organization and cell–cell interactions. Randomly oriented ribbons result in irregular morphology, while aligned ribbons promote elongated cell shapes and enhanced cell–cell alignment along the direction of microribbons. Error bars indicate standard deviation (n ⩾ 10). Statistical analysis in (b) was performed using a two-way ANOVA (factors: *sample type* and *time*) including the interaction term, followed by Tukey’s HSD post-hoc test (*α* = 0.05). Letters above bars denote Tukey groupings within each time point; identical letters with primes (*A, A*′, *A*″) correspond to independent analyses at days 1, 7, and 14, respectively. Groups that do not share a letter are significantly different (*p* < 0.05).

In this study, the adhesion and subsequent proliferation of cells in heterogeneously crosslinked hydrogels were examined using juvenile bovine meniscus cells (JBMCs) as a model and microribbon building blocks with various chemical modifications. Cell attachment and proliferation were assessed by observing cell morphology and counting cell numbers on days 1, 7, and 14 after scaffold fabrication (figures 8(a) and (b)). On each of these days, hydrogel samples were fixed with paraformaldehyde and imaged in 3D using confocal microscopy. To ensure that the JBMCs imaged were located within the interior of the hydrogel, each sample was cut through its center, and images were acquired from the central region of the exposed surface.

On day 1, no statistically significant differences in cell density were observed across hydrogels with different microribbon formulations. By day 7, cell numbers in acetylated (AceA-treated) groups remained largely unchanged, in contrast to the increases observed in other groups. This suggests that acetylation with AceA did not support cell proliferation during this earlier period. This effect is likely due to the reduced surface hydrophilicity caused by acetylation, which may impair protein adsorption and integrin-mediated cell attachment. In contrast, the carboxylation introduced by SucA increases hydrophilicity and may better support cell-spreading and proliferation [37].

By day 14, all groups showed increased cell numbers, likely due to the interconnected pores observed by microscopy, which facilitated cell distribution within the scaffold formed by the microribbon architecture and the large internal surface area that supports three-dimensional cell expansion. The most pronounced increase was observed in groups containing SucA, suggesting that an introduction of carboxylic groups, which enhances hydrogel hydrophilicity and water retention, may have promoted cell survival and proliferation [38]. Notably, the hydrogel with a moderate level of SucA treatment (G-3MAA-2SucA) supported significantly higher cell growth than other SucA-modified groups, suggesting that COOH substitution can promote JBMC proliferation under the tested conditions [39].

Confocal microscope imaging further shows that cells are attached and aligned with the microribbon-formed structures in the heterogeneously crosslinked hydrogel (figure 8(a)). Changing this alignment, such as randomized vs aligned, can therefore control not only the 3D morphology of individual cells but also the alignment among cells, leading to variations in cell morphology, cell–cell contact, cell alignment, and the downstream gene expression that responds to these cell behaviors (figure 8(c)).

Anchorage of cells to the surrounding matrix is a key determinant of success in tissue engineering. Cells actively sense their attachment to the matrix, particularly the strength of adhesion, and respond with specific bioactivities that influence survival, proliferation, and tissue production. This process begins with transmembrane integrin proteins that bind to chemical groups within the extracellular matrix (ECM) [40]. Integrin engagement triggers a cascade of intracellular events, including the assembly of adhesion-associated proteins, which then initiate remodeling of the actin–myosin cytoskeleton. This remodeling involves both the assembly and disassembly of cytoskeletal structures [41]. These dynamic cytoskeletal changes, together with the formation of adhesion complexes, generate intracellular tension. Through actin–myosin contractions, cells continuously exert pulling forces on the ECM while spreading [42].

Our current data cannot resolve whether the observed cell responses are driven primarily by interfacial chemistry or by concomitant changes in matrix mechanics. To address this, future studies will (i) compare MAA-, AceA-, and SucA-modified gelatins synthesized to matched mechanics (similar modulus and degradation kinetics) and (ii) independently vary mechanics while maintaining unchanged chemical composition. Outcomes (viability/proliferation, matrix gene expression, and mechanotransduction readouts) under these two orthogonal designs will help determine whether the extent of carboxylation reflects chemistry-specific effects or mechanics-driven responses.

## 4. Summary

This study introduces a two-step, heterogeneous crosslinking strategy to fabricate structurally defined, highly porous hydrogels using microribbon-shaped gelatin building blocks. In contrast to traditional homogeneous crosslinking methods, which encapsulate cells within tightly meshed networks with sub-micron pore sizes, this approach produces scaffolds composed of microribbons that form interconnected pores and extensive internal surface area at the scale of single cells [3].

When fused via photo-crosslinking, the microribbon building blocks form a continuous hydrogel with tunable three-dimensional architecture and mechanical properties. These heterogeneously crosslinked hydrogels supported cell attachment, spreading, cell–cell alignment, survival, and efficient proliferation in 3D culture, making possible an architecturally guided scaffold design in promoting tissue-engineering [3].

Beyond the use of methacrylic anhydride (MAA) to introduce crosslinkable side chains and provide structural integrity, we also modified the microribbon building blocks with acetic anhydride (AceA) and succinic anhydride (SucA), introducing acetyl and carboxyl groups, respectively. These additional chemical modifications enabled fine-tuning of the hydrogel’s structural and biological performance. At the microscale, AFM measurements revealed that both AceA and SucA reduced microribbon modulus relative to MAA-only controls. Acetylation (via AceA) improved consistency in modulus, possibly by decreasing gelatin solubility and preserving microribbon structure during processing. Carboxylation (via SucA) increased mechanical heterogeneity, likely due to enhanced swelling and erosion, as observed in both AFM and SEM analyses [24, 43–45].

These microscale mechanical changes correlated with differences in cell behavior. Microribbons softened by AceA supported cell survival but did not support proliferation as effectively, possibly due to insufficient modulus or compromised structure. In contrast, moderate carboxylation with SucA (e.g. G-3MAA-2SucA) promoted cell proliferation, likely by balancing swelling, water retention, and matrix integrity.

Chemical modifications also affected degradation profiles. AceA-treated hydrogels degraded faster, consistent with reduced crosslinking density and increased network accessibility. In contrast, SucA slowed degradation, possibly by promoting ionic interactions between carboxyl groups and basic amino acid residues such as lysine, histidine, and arginine. These degradation trends aligned with differences in structural persistence and cell expansion, with SucA-containing hydrogels showing longer-lasting support [46].

Macroscopic mechanical testing further demonstrated that AceA and SucA had distinct effects on bulk hydrogel performance. Tensile tests showed that both modifications decreased tensile modulus and strength, possibly due to reduced availability of MAA for crosslinking. In contrast, compressive testing revealed that moderate carboxylation (G-3MAA-2SucA) enhanced compressive modulus above that of MAA-only controls. This suggests that internal swelling pressure generated by fixed negative charges may counterbalance the lower crosslink density, improving compressive resistance [47]. AceA, by contrast, consistently reduced compressive strength without such compensatory effects.

These findings have important implications for designing tissue-specific hydrogels. Tissues such as tendons and ligaments demand materials with high tensile strength, while cartilaginous tissues require resistance to compressive loads. Such ability to selectively enhance tensile or compressive performance through microribbon organization and chemical functionalization provides a useful strategy for matching scaffold mechanics to target tissues.

In summary, this study establishes a modular, heterogeneous crosslinking platform for hydrogel design. The preparation and use of microribbon-shaped building blocks allows for the creation of 3D scaffolds with defined architecture, tunable chemistry, and tunable mechanics. Chemical modification via side-chain substitution provides further control over the hydrogel’s degradation rate, modulus, and consistency. Together, these capabilities enable the engineering of hydrogels that support and direct cell behavior across cellular and tissue scales, offering a promising approach for next-generation scaffolds in regenerative medicine.

## Data Availability

The data cannot be made publicly available upon publication because no suitable repository exists for hosting data in this field of study. The data that support the findings of this study are available upon reasonable request from the authors.
